# Metabolically healthy obesity reduces the risk of Alzheimer’s disease in elders: a longitudinal study

**DOI:** 10.18632/aging.102496

**Published:** 2019-12-02

**Authors:** Ling-Zhi Ma, Yu-Yuan Huang, Zuo-Teng Wang, Jie-Qiong Li, Xiao-He Hou, Xue-Ning Shen, Ya-Nan Ou, Qiang Dong, Lan Tan, Jin-Tai Yu, Alzheimer’s Disease Neuroimaging Initiative

**Affiliations:** 1 Department of Neurology, Qingdao Municipal Hospital, Qingdao University, Qingdao, China; 2 Department of Neurology and Institute of Neurology, Huashan Hospital, Shanghai Medical College, Fudan University, Shanghai, China

**Keywords:** metabolically healthy obesity, Alzheimer’s disease, elder, amyloid β, cortical structure

## Abstract

A subgroup of overweight/obese individuals, who had favorable metabolic profiles, was termed as metabolically healthy overweight/obese (MHO). Several studies suggested that MHO individuals were not at increased risk of cardiovascular disease and all-course mortality. However, whether MHO is associated with excess risk of Alzheimer’s disease (AD) in elders remains unclear. To explore the risk of AD among MHO phenotype and investigate whether MHO associates with neurodegenerative biomarkers of AD, we assessed body mass index-metabolic status phenotypes of 1199 longitudinal elders from the Alzheimer’s Disease Neuroimaging Initiative (ADNI) cohort using the Adult Treatment Panel-III (ATP- III) criteria. MHO subjects were at a significantly decreased risk for AD (adjusted HR=0.73, 95% CI: 0.54-0.97) compared with metabolically healthy normal weight (MHNW) subjects. In multivariable linear regression models, the cross-sectional associations of MHO with cerebrospinal fluid (CSF) biomarkers, brain Aβ load, and cortical structure were explored. MHO was positively correlated with CSF-Aβ (β=0.746, P=0.015), hippocampal volume (β=0.181, P=0.011), and whole brain volume (β=0.133, P=0.004). The MHO phenotype of the elder conferred a decreased risk of AD and its role may be driven by Aβ.

## INTRODUCTION

Alzheimer’s disease (AD) is the most common type of dementia with an incidence of 60% to 80% [1]. In addition to the recognized risk factors of AD, including age, sex, apolipoprotein *E4* (*APOE Ɛ4*) phenotype, and low education level, the influence of metabolic syndrome (Mets) related factors such as hypertension, type 2 diabetes mellitus (T2DM), dyslipidemia on AD has also begun to be emphasized. Studies have shown that unhealthy metabolic status has a significant impact on brain health and also increases the risk of AD [2–5]. At the same time, several studies showed that obesity at midlife independently increases the risk of AD, while in later life it reduces the risk [6–9].

As overweight/obesity and Mets often coexist, both are used to define the risk of poor health outcomes for overweight/obese individuals and divide subjects into four body mass index-metabolic status phenotype groups: metabolically healthy normal weight (MHNW), metabolically healthy overweight/obese (MHO), metabolically unhealthy normal weight (MUNW), metabolically unhealthy overweight/obesity (MUO) [10]. A subgroup of overweight/obese individuals, who had favorable metabolic profiles such as normal glucose and blood pressure and were free of dyslipidemia, were termed as MHO. Several studies suggested that MHO individuals are overweight/obese but they meet none of the Mets criteria except for body mass index (BMI) or waist circumference [11]. Meanwhile, it has been suggested that MHO individuals were at different risk of AD compared to the MUO group which already has metabolic and obesity problems, although obesity is associated with higher cardiac metabolic risk [12]. Several studies suggested that MHO individuals were not at increased risk of cardiovascular disease and all-course mortality [13, 14]. And MHO individuals showed high insulin sensitivity as well as favorable lipid, inflammation, hormones, liver enzymes and immune status [15]. At present, the study of BMI-metabolic status phenotypes has rarely involved AD. A large-scale population-based cohort study in South Korea has found that MHO subjects had a protective tendency [16]. However, whether MHO is associated with excess risk of AD in the European elderly remains unclear.

In our research, we explored the impact of the MHO phenotype on the development of AD in the elderly. Apart from that, we wonder if the effects of overweight/obesity on elders with different metabolic states are consistent since MUO subjects did not show a protective tendency in previous studies [16]. Furthermore, we make the assumption that BMI-metabolic status phenotypes have differences in their AD-related biomarkers to explore possible mechanisms whereby MHO affects the occurrence of AD.

## RESULTS

### Baseline characteristics of the study population

Table 1 shows the baseline characteristics of the participants included in the analysis, results of post hoc multiple comparisons are shown in Supplementary Table 1. The mean age was 73.5 ± 7.1 years and the mean BMI was 27.0 ± 4.7 kg/m^2^. Among the 1199 participants, 495 (41.3%) were classified as metabolically unhealthy. The MHO group was the youngest (72.8 ± 7.3 years). The metabolically unhealthy groups have higher blood pressure, fasting glucose, triglyceride (TG), and low-density lipoprotein cholesterol (LDL-C) levels, while high-density lipoprotein cholesterol (HDL-C) was higher in the metabolically healthy groups. In terms of medical history, the MUO group showed the highest proportion of hypertension, T2DM, and previous myocardial infarction.

**Table 1:** Baseline characteristics of study participants according to body mass index-metabolic status phenotypes.

**Characteristic**	**Metabolically healthy(n=704)**		**Metabolically unhealthy(n=495)**		**P Value**
	**MHNW (n=289)**	**MHO(n=415)**	**MUNW (n=132)**	**MUO (n=363)**	
Age, y	73.5±7.4	72.8±7.3	76.5±6.3	73.2±6.7	<0.001
M/F, n	146/143	242/173	57/75	227/136	<0.001
Formal education, y	16.6±2.7	16.0±2.8	16.0±2.8	15.7±2.8	0.002
Cognitive diagnosis					
Normal control	89(30.8)	138(33.3)	55(41.7)	120(33.1)	
Mild cognitive impairment	200(69.2)	277(66.7)	77(58.3)	243(66.9)	0.176
*APOEε4* Carriers	142 (49)	157 (36)	54 (41)	157 (43)	0.028
Weight, kg	65.9±9.9	82.1±12.0	65.3±9.7	86.2±15.1	<0.001
Height, cm	169.9±10.2	168.9±10.3	168.9±11.3	169.5±10.5	0.590
BMI, kg/m^2^	22.7±1.6	28.8±3.7	22.8±1.7	30.0±4.4	<0.001
Systolic BP, mmHg	130.7±18.0	132.9±15.8	142.0±17.2	139.5±15.4	<0.001
Diastolic BP, mmHg	73.3±9.9	75.6±9.8	74.2±10.0	76.3±9.1	<0.001
Fasting glucose, mg/dl	93.0±13.9	93.6±13.8	110.8±28.2	113.4±32.4	<0.001
Triglyceride, mmol/L	1.0±0.3	1.1±0.4	1.2±0.5	1.3±0.5	<0.001
HDL-C, mmol/L	1.6±0.3	1.5±0.3	1.5±0.4	1.3±0.3	<0.001
LDL-C, mmol/L	1.4±0.4	1.3±0.4	1.4±0.4	1.3±0.4	<0.001
Smoker	84(29.1)	135(32.5)	35(26.5)	100(27.5)	0.380
Drinker	10(3.5)	13(3.1)	3(2.3)	10(2.8)	0.910
Medical history					
Hypertension	85(29.4)	159(38.3)	81(61.4)	241(66.4)	<0.001
T2DM	5(1.7)	8(1.9)	23(17.4)	84(23.1)	<0.001
Previous myocardial infarction	13(4.5)	28(6.7)	3(2.3)	32(8.8)	0.190

Values are mean±standard deviation(SD), or n (% of the group).

Abbreviations: MHNW, metabolically healthy normal weight; MHO, metabolically healthy overweight/obese; MUNW, metabolically unhealthy normal weight; MUO, metabolically unhealthy overweight/obese; M, male; F, female; *APOEε4*, apolipoprotein E4; BMI, body mass index; BP, blood pressure; HDL-C, high-density lipoprotein cholesterol; LDL-C, low- density lipoprotein cholesterol; T2DM, type 2 diabetes mellitus.

### Risk of incident AD according to different BMI-metabolic status phenotypes

A total of 321 (26.8%) participants developed AD during the follow-up. It is worth noting that the MHO group showed significantly lower AD incidence (21.7%) compared with the MHNW reference group (30.8%). As expected, compared with the normal weight group, the overweight/obese group were at a reduced risk of incident AD (HR=0.70, 95% CI: 0.56-0.89), after adjustment for age, sex, *APOE Ɛ4* status, cognitive diagnosis, education, tobacco and alcohol use, LDL-C and metabolic status. Risk in the metabolically unhealthy group (HR=1.28, 95% CI: 1.02-1.60) was 1.28 times higher than that in the metabolically healthy group after further adjustment for BMI status.

The Kaplan-Meier curves for cumulative survival free from, incident AD as a function of BMI-metabolic status phenotypes showed that these survival curves differ significantly from each other over the follow-up (log-rank, P = 0.02). Unadjusted and adjusted HRs and 95% CIs of AD compared with MHNW group, according to BMI-metabolic status phenotypes are listed in Table 2.

**Table 2:** HRs of Alzheimer’s disease according to body mass index-metabolic status phenotypes.

	**MHNW**	**MUNW**	**MHO**	**MUO**
Model1*	1(ref)	1.09(0.75-1.57)	0.67(0.50-0.90)	0.89(0.65-1.15)
Model2†	1(ref)	1.22(0.84-1.78)	0.73(0.54-0.97)	0.93(0.70-1.24)
Model3‡	1(ref)	1.22(0.84-1.78)	0.73(0.54-0.97)	0.93(0.70-1.24)

Abbreviations: MHNW, metabolically healthy normal weight; MUNW, metabolically unhealthy normal weight; MHO, metabolically healthy overweight/obese; MUO, metabolically unhealthy overweight/obese.

*Unadjusted

†Adjusted for age, sex, *APOEε4* status and cognitive diagnosis.

‡Further adjusted for education, tobacco and alcohol use and low-density lipoprotein.

**Figure 1:**
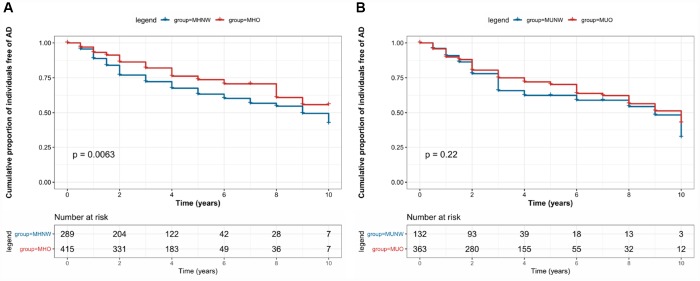
**Kaplan–Meier survival curves showing the associations between body mass index-metabolic status phenotypes and the risk of Alzheimer’s disease.**

**Figure 2:**
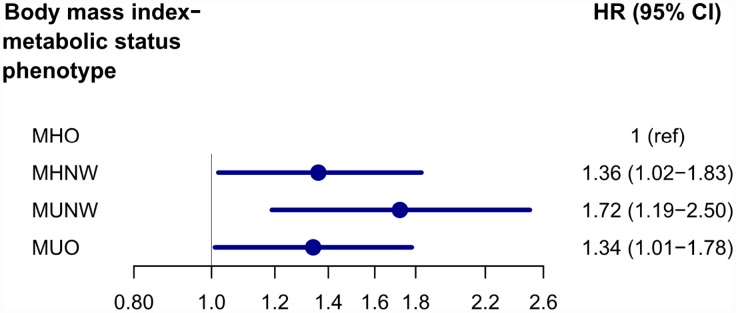
Adjusted* HRs and 95% Confidence Intervals of MHNW, MUNW and MUO groups with MHO group as reference. *Adjusted for age, sex, *APOE Ɛ4* status, cognitive diagnosis, education, tobacco and alcohol use, and low-density lipoprotein cholesterol. Abbreviations: MHNW, metabolically healthy normal weight; MUNW, metabolically unhealthy normal weight; MHO, metabolically healthy overweight/obese; MUO, metabolically unhealthy overweight/obese.

We present the association between BMI and incident AD in analysis stratified by metabolic status category; the metabolically healthy group was the reference within each strata of BMI. Figure 1 shows the Kaplan-Meier curves for the cumulative proportion of individuals free of AD, in which MHO group had a lower probability of developing AD compared to MHNW group (log rank P=0.006), while the metabolically unhealthy group did not show this trend (log rank P=0.22). There was still a significant difference in AD risk between metabolically healthy groups after multivariable adjustment: compared with the MHNW group, the MHO group had a lower risk for AD (adjusted HR=0.73, 95% CI: 0.54-0.97, Model3). However, there was no significant difference between metabolically unhealthy groups after multivariable adjustment: compared with the MUNW group, the MUO group did not show a different risk (unadjusted HR=0.80, 95% CI: 0.56-1.16, Model1; adjusted HR= 0.75, 95% CI: 0.52-1.09, Model2; adjusted HR=0.75, 95% CI: 0.52-1.09, Model3). In addition, compared with MHO group, MHNW (adjusted HR=1.36, 95% CI: 1.02-1.83), MUNW (adjusted HR=1.72, 95% CI: 1.19-2.50), and MUO (adjusted HR=1.34, 95% CI: 1.01-1.78) groups all showed significant higher AD risks after adjustment for same factors as previous studies (Figure 2).

### BMI-metabolic status phenotypes and AD-related biomarkers

**Figure 3:**
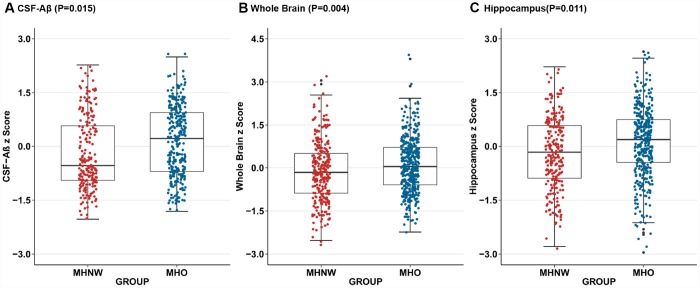
**Adjusted for age, sex, *APOE Ɛ4* status, cognitive diagnosis, education, tobacco and alcohol use, and low-density lipoprotein cholesterol, compared to MHNW group, MHO group had higher CSF-Aβ concentrations(P=0.015)** (**A**) on the baseline as well as larger whole brain volumes(P=0.004) (**B**) and hippocampal volumes (P=0.011) (**C**) after additional correction for the intracranial volume. Abbreviations: MHNW, metabolically healthy normal weight; MHO, metabolically healthy overweight/obese; CSF, cerebrospinal fluid; Aβ, Amyloid β.

As for metabolically healthy groups, our results indicated that the MHO group was associated with higher cerebrospinal fluid (CSF)-Amyloid β (Aβ) (β=0.746, P=0.015) (Figure 3A) compared with the MHNW group. No statistical differences were observed in CSF total tau (t-tau) and phosphorylated tau (p-tau) between the two groups. There were differences in several brain regions of interest (ROIs) (whole brain, hippocampus, entorhinal, and middle temporal) between MHO and MHNW group in the unadjusted model. It is worth noting that the correlations of MHO with larger volume of whole brain (β=0.133, P=0.004) (Figure 3B) and hippocampus (β=0.181, P=0.011) (Figure 3C) remained significant in multiple linear regression analyses adjusting for age, sex, APOE status, cognitive diagnosis, education, tobacco and alcohol use, LDL and intracranial volume. All these associations still achieved significance after false discovery rate (FDR) correction. No difference was detected between MUO and MUNW groups in cortical thickness and florbetapir cortical standardized uptake values ratios (SUVRs).

As for metabolically unhealthy groups, we did not observe any difference in CSF biomarkers between MUNW and MUO groups. However, in the unadjusted linear regression model, MUO group presented a correlation with higher volumes of whole brain (β =0.000, p = 0.002), hippocampus (β = 0.038, p = 0.001), and entorhinal (β = 0.019, p = 0.012) compared with MUNW group, which failed to reduplicate in further analysis adjusted for age, sex, *APOE Ɛ4* status, cognitive diagnosis, education, tobacco and alcohol use, LDL and intracranial volume (whole brain: β =0.028, p=0.028 and hippocampus: β =0.144, p =0.117 and entorhinal: β =0.209, p=0.041). Similarly, no difference was detected between MUO and MUNW groups in cortical thickness and SUVRs. Detailed unadjusted and adjusted β coefficients and p-values in four subgroups were listed in Supplementary Table 2.

## DISCUSSION

In this longitudinal study of 1199 individuals initially free of AD, 321 developed AD during an average follow-up. Previous studies investigating the associations of AD with obesity and unhealthy metabolic profiles reported that late-life obesity reduced the risk and metabolically unhealthy individuals had an increased risk. Our results showed that after correcting for the metabolic state, the risk of AD in elderly obese individuals was significantly reduced (HR=0.70, 95% CI: 0.56-0.89), and the risk of metabolic abnormalities increased after correcting for the obese status (HR=1.28, 95% CI: 1.02-1.60), which is in line with the results of previous studies [17]. In addition, we analyzed the risk of incident AD according to late-life BMI-metabolic status phenotypes. The results showed that MHO individuals had a lower risk for AD compared with the MHNW group. This protective tendency was not observed in MUO individuals compared with the MUNW group. A large-scale population-based cohort study previously reported the association between BMI-metabolic status phenotypes and the risk of AD. It showed an increased risk of AD in the MUO group, which was inconsistent with our results [16]. However, the effect of *APOE Ɛ4* status has not been corrected for in this study. We had carefully adjusted for most confounding variables, especially *APOE Ɛ4* status which had a significant impact on the risk of AD. Therefore, we believed that the risk of AD in MUO group cannot be fully determined and deserves further population studies.

Most previous studies on obesity did not separate metabolically healthy individuals from metabolically unhealthy individuals [18]. However, the effects of metabolic status cannot be ignored, because obesity and unhealthy metabolic states can exist independently, which means that it is necessary to study the effects of obesity on AD in different metabolic states [10]. Our results broke the “obesity paradox” to some extent, in which overweight and obese individuals are pretending to have lower mortality in various established studies [19–21]. Therefore, the emphases on the influence of metabolic status and early management of high BMI elder individuals with metabolic abnormalities may be beneficial in the prevention of AD events.

In this study, we further examined the relationship between BMI-metabolic status phenotypes and AD-related biomarkers in this non-dementia sample. We now found MHO was positively associated with CSF-Aβ pathology and this association remained significant after controlling for several possible confounders. We did not find evidence for a significant relationship between MHO and tau pathology in the CSF. Our study also demonstrated that MHO showed suggestive associations with hippocampal and whole brain volumes. We did not find associations between MUO individuals and any AD-related biomarkers. The results of our study also suggested a likely mechanism of overweight/obesity in preventing the onset of AD dementia when metabolism is normal. Although Aβ plays an upstream role in the pathogenesis of AD, Aβ alone is insufficient to cause the onset of clinically detected dementia directly. However, this may be sufficient to cause downstream pathophysiologic changes such as brain atrophy that ultimately led to AD dementia [22]. We speculated that in metabolically healthy individuals, overweight/obesity influenced Aβ pathology first, and subsequently reduced downstream brain atrophy rate and the risk of AD dementia.

There are many possible mechanisms that explain the beneficial effects of MHO on the development of AD dementia. A decline in the levels of plasma insulin-like growth factor I (IGF-1), which has been shown to be an independent risk factor for AD [23], was observed in the underweight group [24]. IGF-1 exerted neurotrophic effects on the hippocampus, and high levels of IGF-1 were associated with better cognitive performance [25]. In addition, certain adipokines secreted by adipose tissue, such as leptin, may play a key role [26]. A higher circulating leptin level is associated with better cognitive performance, a lower incidence of AD dementia and larger volumes of brain regions, such as the hippocampus [18, 27]. The results of previous studies also indicated that MHO individuals have fewer years of obesity than their MUO counterparts [28]. Evidence showed that MHO individuals had certain advantages in metabolism and other aspects compared with the MUO population, which may affect the occurrence of AD. Results from previous studies suggested that MUO individuals have been overweight/obese for longer years compared to MHO individuals [28]. Despite having a high accumulation of body fat, MHO individuals display lower levels of C-reactive protein [29], higher adiponectin concentrations [30], higher insulin sensitivity [31], and a lower risk of type 2 diabetes. Therefore, the estimation of AD risk in elderly obese people cannot ignore the impact of different metabolic statuses.

It is worth noting that this study has some limitations. First, BMI or metabolic status in a certain proportion of participants will change over time, but our study did not reflect longitudinal changes in body weight or laboratory results. Second, current research on AD-related biomarkers is based on cross-sectional data, which do not represent individual longitudinal changes. Third, the study is limited to the ADNI cohort, which is not a population-based cohort. Nevertheless, studies including larger samples are needed to further examine the effects of MHO.

In conclusion, we showed different risks of AD in elderly overweight/obese individuals with different metabolism statuses. The MHO phenotype conferred a lower risk of AD when compared with MHNW or MUO. The protective effect of overweight/obesity in the presence of metabolic abnormalities was not apparent compared with MUNW. Metabolic abnormalities in the elderly should not be neglected. Further studies in other populations are warranted, to better understand the effects of metabolic status and obesity on AD.

## MATERIALS AND METHODS

### Participants

Data used in this study was obtained from the ADNI database (adni.loni.usc.edu), which was launched in 2003 as a public-private partnership led by Principal Investigator Michael W. Weiner, MD. The primary goal of ADNI has been to test whether serial MRI, PET, other biological markers, and clinical and neuropsychological assessments can be combined to measure the progression of MCI and early AD [32]. Regional ethical committees of all participating institutions approved the ADNI. Individuals were excluded if they had Hachinski ischemic score > 4 (a high risk of cerebrovascular disease contributing to cognitive impairment), were unable to undergo MRI, had other neurologic disorders, active depression, history of psychiatric diagnosis, alcohol or substance dependence in the last 2 years, had less than 6 years of education, or were not fluent in English or Spanish [33]. All study participants provided written informed consent. For up-to-date information, see www.adni-info.org.

A total of 1199 individuals (normal controls [CN]=402, mild cognitive impairment [MCI]=797 at baseline) with an average age of 73.5 years, and at least 6 years of education were included in this study. Those with severe cerebrovascular disease have been excluded before being enrolled in the ADNI. We excluded participants whose data on baseline BMI, metabolic status phenotypes were missing. For the current analysis, underweight (BMI < 18.5 kg/m^2^) participants (n=9) were excluded.

### Measurement of laboratory and anthropometric parameters

All data on laboratory and anthropometric parameters, as well as medical history are downloaded from the ADNI database. Calibrated mercury sphygmo-manometer and blood pressure cuff are used to measure arterial blood pressure. During the blood pressure measurement, participants were seated with their forearms placed horizontally in the four rib spaces of the sternum (i.e. the level of the heart) [34]. TG, HDL-C, and LDL-C, and fasting venous blood glucose were measured. BMI was calculated by dividing body weight (in kg) by height squared (in m^2^). In the *APOE Ɛ4* genotyping performed at the ADNI Biomarker Core Laboratory (University of Pennsylvania), participants carrying at least one allele were identified as *APOE Ɛ4* positive status [35]. We identified the baseline comorbidities, including hypertension, T2DM, and previous myocardial infarction, by screening the medical information database. Self-reported health behaviors included smoking status (categorized as yes or no, both previous and current smokers are identified as yes) and alcohol consumption which is grouped in the same way.

### Definitions of BMI-metabolic status phenotypes

We used standard operating protocols to measure ATP-III components to define metabolic status [36]. Participants who met ≥2 of the following four parameters were defined as metabolically unhealthy : 1) elevated systolic blood pressure (≥130 mmHg) or diastolic blood pressure (≥85 mmHg) or anti-hypertensive treatment; 2) elevated fasting plasma glucose (≥ 100 mg/dL) or anti-diabetic treatment; 3) elevated TG (≥1.7 mmol/L); 4) reduced HDL-C (<1.0 mmol/L for men and <1.3 mmol/L for women). BMI was used to determine obese phenotypes according to world health organization criteria: overweight/obese ≥25 kg/m^2^, normal weight <25 kg/m^2^ [37]. The waist circumference criterion was not used because of its collinearity with BMI.

Participants were categorized into the following groups: 1) MHNW: BMI < 25 kg/m^2^ and <2 metabolic risk factors; 2) MHO: BMI ≥ 25kg/m^2^ and <2 metabolic risk factors; 3) MUNW: BMI < 25 kg/m^2^ and ≥2 metabolic risk factors; 4) MUO: BMI ≥ 25 kg/m^2^ and ≥2 metabolic risk factors.

### Definition of incident AD

The primary endpoint was newly diagnosed AD during the follow-up period. AD patients were required to meet the criteria for probable AD defined by the National Institute of Neurological and Communicative Disorders and Stroke–Alzheimer’s Disease and Related Disorders Association [38]. In brief, AD group had a Mini-Mental State Examination (MMSE) score of 20-26 where lower scores suggest severe cognitive impairment (range, 0–30), and a Clinical Dementia Rating (CDR) score of 0.5 to 1.0 where lower scores indicate mild cognitive impairment (range, 0–3) [33]. Participants without AD during the follow-up period were classified as missing or not having observed outcomes, depending on the circumstances.

### CSF data

A multiplex xMAP Luminex platform (Luminex Corp, Austin, TX) with Innogenetics (INNO-BIA AlzBio3, Ghent, immunoassay kit-based reagents Belgium; for research use only reagents) was used to collect and analyze samples, including CSF Aβ42, CSF t-tau, and CSF p-tau (pg/mL). Details have been described previously [39].

### Neuroimaging data

The neuroimaging data, such as regional volume, cortical thickness on MRI and SUVRs via F18-PET-AV45 were all downloaded from the ADNI dataset. Preprocessed data are available online (adni.loni.ucla.edu/about-data samples/image-data/). Details on neuroimaging methods utilized by ADNI have been described previously [40]. In brief, FreeSurfer (version 5.1) was used to extract volumetric and cortical thickness data after correcting the scans. After removing the non-brain tissue using a hybrid watershed/surface deformation, automated Talairach transformation was used for segmentation of subcortical white matter and deep gray matter volume structures [41]. For F18-PET-AV45, preprocessed florbetapir image data and co-registered structural magnetic resonance images were analyzed using Freesurfer software, version 4.5.0 (surfer.nmr.mgh.harvard.edu/) as described online (adni.loni.ucla.edu/research/ pet-post-processing/). The SUVRs, measured by the florbetapir AV45 standardized uptake value ratio, was normalized to the whole cerebellum as a summary measure of florbetapir retention for each participant [42]. In our study, we used differences in baseline data (ROIs, cortical thickness and SUVRs) in interesting regions such as the hippocampus, and entorhinal cortex, which had established roles in predicting AD risk and progression, to calculate differences in BMI-metabolic status phenotypes across the AD spectrum.

### Statistical analysis

We used the Chi-Square test to explore the differences between baseline characteristics of categorized variables and used variance (ANOVA) to explore those for continuous variables. And performed post hoc multiple comparisons among four groups. The cumulative AD incidence for each group was plotted with Kaplan-Meier curves and the effects of obesity in normal and abnormal metabolic groups were compared by the log-rank test. Multivariable Cox proportional hazards regression analysis was performed to assess the risk of new-onset AD with different BMI-metabolic status phenotypes in 3 models: unadjusted model 1, model 2 adjusted for age, sex, *APOE Ɛ4* status and cognitive diagnosis, and model 3 further adjusted for education, tobacco and alcohol use, and LDL-C. For each individual, the baseline time was defined by the ADNI database, and two endpoints were chosen: the time of AD diagnosis or the last follow-up time of participants. AD risk was expressed as the HR with 95% CI. In the first set of analyses, we used the MHNW group as the reference. We then ran a second set of analyses, stratified by metabolic category to compare the risk of AD as a function of BMI status in each category, the normal weight group, within each metabolic category, was the reference in these analyses.

To examine the effects of BMI-metabolic status phenotypes on the chosen AD-related biomarkers, we evaluated the association between BMI-metabolic status phenotypes and these various biomarkers using multiple linear regression models at baseline. These models assumed a random subject-specific intercept and a random subject-specific slope. Since all outcome variables were converted to normalized Z-scores, β coefficients refer to standardized effects. All tests were 2-sided and the criterion for statistical significance was p < 0.05 according to FDR correction. All regression analyses were adjusted for age, sex, *APOE Ɛ4* status, cognitive diagnosis, education, tobacco and alcohol use, LDL-C, and intracranial volume or reference region. All statistical analyses were performed using a software program (R 3.5.1).

## Supplementary Material

Supplementary Tables
