# Supplementary material for: Metabolically healthy obesity reduces the risk of Alzheimer’s disease in elders: a longitudinal study
Source: Aging (Albany NY). 2019 Dec 2;11(23):10939–51. doi: 10.18632/aging.102496 (PMC6932886; doi:10.18632/aging.102496)
Supplement: Supplementary Tables [file aging-11-102496-s001..pdf]

SUPPLEMENTARY TABLES

Supplementary Table 1. Baseline characteristics of study participants according to body mass index-metabolic status phenotypes (Post Hoc Multiple Comparison).

Characteristic	MHNW vs MHO (P value)	MHNW vs MUNW (P value)	MHNW vs MUO (P value)	MHO vs MUNW (P value)	MHO vs MUO (P value)	MUNW vs MUO (P value)
Age	0.522	<0.001	0.957	<0.001	0.808	<0.001
M/F	0.041	0.162	0.002	0.002	0.230	<0.001
Formal education	0.035	0.169	0.001	1.000	0.537	0.825
Cognitive diagnosis	0.493	0.029	0.539	0.078	0.954	0.076
APOEε4 Carriers	0.003	0.116	0.134	0.527	0.124	0.641
Weight	<0.001	0.959	<0.001	<0.001	<0.001	<0.001
Height	0.578	0.833	0.968	1.000	0.826	0.955
BMI	<0.001	0.988	<0.001	<0.001	<0.001	<0.001
Systolic BP	0.310	<0.001	<0.001	<0.001	<0.001	0.432
Diastolic BP	0.012	0.827	<0.001	0.475	0.700	0.129
Fasting glucose	0.981	<0.001	<0.001	<0.001	<0.001	0.678
Triglyceride	0.007	<0.001	<0.001	0.042	<0.001	<0.001
HDL-C	<0.001	0.009	<0.001	0.460	<0.001	<0.001
LDL-C	0.441	0.820	<0.001	0.996	0.011	0.077
Smoker	0.329	0.590	0.669	0.193	0.131	0.819
Drinker	0.810	0.513	0.604	0.610	0.756	0.767
Medical history						
Hypertension	0.015	<0.001	<0.001	<0.001	<0.001	0.300
T2DM	0.848	<0.001	<0.001	<0.001	<0.001	0.172
Previous myocardial infarction	0.210	0.268	0.031	0.053	0.281	0.012

Abbreviations: MHNW, metabolically healthy normal weight; MHO, metabolically healthy overweight/obese; MUNW, metabolically unhealthy normal weight; MUO, metabolically unhealthy overweight/obese; M, male; F, female; *APOEε4*, apolipoprotein E4; BMI, body mass index; BP, blood pressure; HDL-C, high-density lipoprotein cholesterol; LDL-C, low-density lipoprotein cholesterol; T2DM, type 2 diabetes mellitus.

Supplementary Table 2. Unadjusted and adjusted association of body mass index-metabolic status phenotypes With CSF Aβ/t-tau, ROIs, cortical thickness and PET SUVRs.

	MHO/MHNW				MUO/MUNW			
	unadjusted		adjusted		unadjusted		adjusted	
	β	P	β	P	β	P	β	P
**CSF***								
CSF Aβ	1.340	**0.000**	0.746	**0.015**	0.033	0.362	0.021	0.540
CSF t-tau	-0.027	0.023	-0.015	0.189	-0.019	0.206	-0.006	0.668
CSF p-tau	-0.016	0.565	0.011	0.686	0.001	0.983	0.026	0.652
**ROIs†**								
Whole Brain	0.021	**0.000**	0.133	**0.004**	0.000	**0.002**	0.028	0.626
Hippocampus	0.032	**0.000**	0.181	**0.011**	0.038	**0.001**	0.144	0.117
Entorhinal	0.015	**0.010**	0.126	0.099	0.019	**0.012**	0.209	0.041
Middle Temporal	0.055	**0.009**	0.127	0.071	0.042	0.128	0.069	0.465
**Cortical thickness***								
Left Parahippocampus	0.163	0.103	0.153	0.144	0.018	0.735	0.004	0.975
Right Parahippocampus	0.099	0.276	0.095	0.362	0.110	0.302	0.166	0.236
Left Entorhinal	0.462	0.336	0.077	0.431	0.604	0.101	0.115	0.387
Right Entorhinal	0.866	0.379	0.167	0.092	0.387	0.244	0.137	0.309
Left Middle Temporal	0.503	0.255	0.015	0.878	1.010	0.072	0.022	0.867
Right Middle Temporal	0.796	0.037	0.004	0.967	1.061	0.057	-0.023	0.861
**PET SUVRs‡**								
Left Hippocampus	0.006	0.512	0.052	0.407	0.003	0.326	-0.053	0.511
Right Hippocampus	0.005	0.583	0.042	0.494	0.014	0.390	-0.009	0.924
Left Entorhinal	-0.011	0.337	-0.019	0.809	-0.004	0.819	0.015	0.902
Right Entorhinal	-0.008	0.486	-0.015	0.850	-0.004	0.826	0.056	0.651
Left Middle Temporal	-0.027	0.073	-0.008	0.918	-0.000	0.994	0.067	0.582
Right Middle Temporal	-0.033	0.024	-0.053	0.511	0.004	0.821	0.128	0.287

Adjusted P values are listed in the table.
*Adjusted for age, sex, *APOE Ɛ4* status, cognitive diagnosis, education, tobacco and alcohol use and low-density lipoprotein.
† Adjusted for age, sex, *APOE Ɛ4* status, cognitive diagnosis, education, tobacco and alcohol use, low-density lipoprotein and intracranial volume.
‡ Adjusted for age, sex, *APOE Ɛ4* status, cognitive diagnosis, education, tobacco and alcohol use, low-density lipoprotein and reference region.
The unadjusted P values of ROIs are calculated from the data corrected by intracranial volume, and the unadjusted P values of PET SUVRs are calculated from the data corrected by reference region.
Bonferroni corrected P values <0.05 have been marked
Abbreviations: MHNW, metabolically healthy normal weight; MHO, metabolically healthy overweight/obese; MUNW, metabolically unhealthy normal weight; MUO, metabolically unhealthy overweight/obese.
